# The Promising Role of Chemokines in Vitiligo: From Oxidative Stress to the Autoimmune Response

**DOI:** 10.1155/2022/8796735

**Published:** 2022-01-19

**Authors:** Shan He, Jinhua Xu, Jinfeng Wu

**Affiliations:** Department of Dermatology, Huashan Hospital, Fudan University, Shanghai 200040, China

## Abstract

Vitiligo is a common chronic autoimmune skin disorder featured with depigmented patches and underlying destruction of melanocytes in the lesional skin. Multiple factors and mechanisms have been proposed for the etiopathogenesis of vitiligo, among which oxidative stress has been widely accepted as a key factor in initiating melanocyte loss. The altered redox status caused by oxidative stress, including the overproduction of reactive oxygen species (ROS) and the decreased activity of the antioxidant system in the skin, surrenders the resistance of melanocytes to exogenous or endogenous stimuli and eventually impairs the normal defense mechanism, leading to the absence of melanocytes. Considering the important role of innate and adaptive immunity in vitiligo, there is mounting evidence revealing an association between oxidative stress and autoimmunity. Since the significant changes of chemokines have been documented in vitiligo in many recent studies, it has been suggested that ROS-mediated chemotactic signals are not only the biomarkers of disease progression and prognosis but also are involved in the pathogenesis of vitiligo by facilitating the innate and adaptive immune cells, especially melanocyte-specific T cells, trafficking to the lesional areas of vitiligo. In this review, we discuss the interaction between oxidative stress and autoimmune response orchestrated by chemokines, including CXCL16-CXCR6 axis, CXCL9/CXCL10-CXCR3 axis, and other altered chemokines in vitiligo, and we also try to provide insight into potential therapeutic options through targeting these pathways.

## 1. Introduction

Vitiligo is an acquired, chronic skin disorder characterized by epidermal melanocyte loss and the clinical appearance of patchy depigmentation on the skin. Although it only affects approximately 1% of the world's population, appearance alternations and psychological stress can lead to impaired life quality in patients [1].

The current studies for vitiligo etiopathogenesis reveal the involvement of multiple mechanisms, including oxidative stress, metabolic disorder, and autoimmune response [1]. Various triggers, ranging from mechanical stimulus to chemical exposures, are correlated with the onset of vitiligo. These triggers are considered to induce stress responses in keratinocytes and melanocytes, leading to the imbalance of the oxidative and antioxidant systems. Stressed melanocytes and keratinocytes can produce a higher amount of proinflammatory cytokines and chemokines to form an aberrant epidermal microenvironment, and the damaged melanocytes release self-antigens and immunostimulatory signals [2]. Under the overproduction of ROS, as the major agents of oxidative stress, the induced antigen release can be sensed and processed by antigen-presenting cells (APCs) such as local dendritic cells (DCs), ultimately resulting in increased infiltration of melanocyte-specific T cells in perilesional and lesional skin and the production of antibodies against melanocytes to activate the adaptive immune response in the vitiligo epidermis [2–5]. One of the crucial events in such autoimmune response is immune cell migration and retention following the chemotactic signals [2].

The chemokines that regulate these chemotactic signals are small molecules produced by both resident cells and infiltrated cells in the skin. While the chemotactic effect of chemokines has been known for many years, accumulating evidence indicates the broader functions of chemokines as critical factors in both normal functions of the immune system and pathogenesis of many inflammatory or autoimmune disorders, including neoangiogenesis and organogenesis [6, 7]. The increased levels of some chemokines, such as CXCL10 and CXCL8, in the serum and epidermis of vitiligo have been reported in many recent studies [6, 8]. Genetic and proteomics studies on vitiligo provide indirect evidence for the important role of chemokines in vitiligo [9]. Considering the imbalanced oxidative status in autoimmune diseases, the association between oxidative stress and chemokines has been documented in some diseases, such as systemic lupus erythematosus and rheumatoid arthritis [7]. Although early analysis of chemokines in vitiligo indicated that the expression of chemokines, such as CXCL9 and CXCL10, could be reliable biomarkers for disease activity and prognosis, emerging studies have shed light on the interaction between the increased ROS levels and levels of several chemokines, such as CXCL16 and CXCL10, to act as key effectors in the initiation and progression of vitiligo [10]. These ROS-induced chemokines further mediate the migration of immune cells, especially T cells, in the lesion of vitiligo patients [10, 11].

In this review, we not only focus on the sources and expressions of these chemokines in the context of vitiligo but also reveal the functional roles and underlying mechanisms involved in the interaction between oxidative stress and autoimmunity in the pathogenesis and progression of vitiligo, which might further provide insight into potential treatment approaches for vitiligo patients.

## 2. The Chemokine Superfamily and Homeostatic Chemokine Signals in the Skin

Chemokines (chemotactic cytokines) are a superfamily of small (8-12 kDa), secreted proteins, which are best known for their regulation of cell migration, especially leukocytes. Based on the number and sequence of N-terminal cysteine residues, chemokines can be categorized into four different families: CXC, CC, CX3C, and XC chemokines. Chemokine receptors are typically G protein-coupled seven-transmembrane receptors, named as CXC receptor (CXCR), CC receptor (CCR), CX3C receptor (CX3CR), and XC receptor (XCR) correspondingly. There are now approximately 50 endogenous chemokine ligands and 20 typical G protein-coupled receptors. Chemokines can also bind to atypical G protein-independent chemokine receptors to regulate chemokine gradient [12].

Homeostatic chemokines that are recognized by chemokines produced constitutively have long been a significant feature to recruit and retain cells in normal or steady-state skin, especially cells for immune surveillance. A vast majority of skin resident cells can be sources of homeostatic chemokines to facilitate immune cell recruitment and retention in the skin, among which keratinocytes are a major source of chemokines, with the infundibulum and the suprabulb expressing CXCL4, CXCL9, CXCL10, CCL1, and CCL8 at relative high levels [13, 14]. In addition, CCL20 expressed exclusively by the hair follicle (HF) epithelium in neonatal skin can mediate the migration of CCR6-positive neonatal regulatory T (Treg) cells, and Treg cells, in turn, facilitate immune homeostasis in these tissues [15]. Treg cells that reside near the HF can further regulate the CXCL5-T-helper (Th) 17-neutrophil axis to promote hair follicle stem cells (HFSC) migration and the egress of keratinocytes derived from HFSC, especially during the repairment of the epidermal barrier [16].

While among the immune cells that interspersed the epidermal and dermal layers, skin resident T cells express CCR4, CCR8, CXCR6 and CCR6 at a high level [14, 17]. CCR4 and CCR6 on immune cells can assist homeostatic T cell trafficking to the skin [14, 18, 19]. CXCR6 expressed mainly by T cells and NK cells exerts its effects through its cognate ligand CXCL16 to maintain the immune cell compartment in the skin [14]. CXCR6 expression also helps the retention of skin resident memory T cells. Due to an upregulated level of CXCL16 in skin diseases, the role of the CXCL16-CXCR6 axis in inflammation is under investigation recently [14, 20]. Skin resident T cells lacking CCR8 exhibit changes associated with the activation and effector functions of T cells, such as increased expression of inflammatory chemokine receptor CXCR3 and promoted gene coding cytolytic molecule perforin [21]. However, the absence of CCR8 did not influence the recruitment of T cells against skin infection, indicating that CCR8 may be more essential in the homeostasis in the skin [20].

## 3. Interaction between Chemokines and Oxidative Stress in the Development of Vitiligo

**Figure 1:**
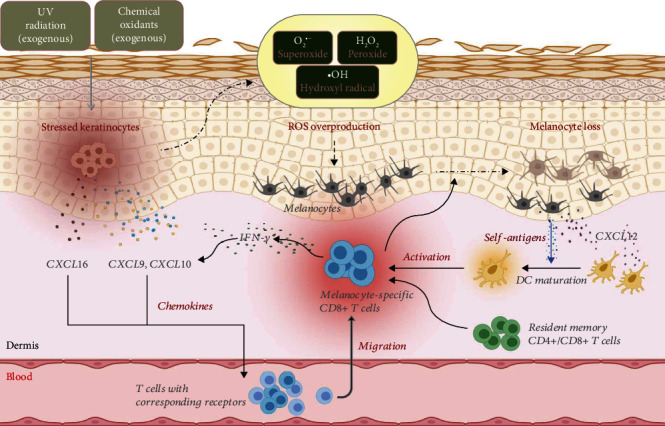
The interaction between oxidative stress and autoimmunity through the chemotactic signals in vitiligo.

The regulation of endogenous ROS production and its balance with the antioxidant system is dynamic and complex. Under physiological conditions, mild stress such as moderate sun exposure or physical exercise can induce the release of ROS to activate response for the maintenance of cellular defense barrier function [22]. Given the importance of ROS production during skin injury, repair, regeneration, and aging processes, many studies have linked oxidative stress with various inflammatory skin diseases through triggering chemokines and cytokines to provoke the autoimmune response [23]. From the onset and subsequent autoimmune response of vitiligo, oxidative stress has been considered an initial event and an essential factor, which can be supported by the overproduction of ROS in both lesional and nonlesional skin [10]. The increased levels of ROS can be attributed to multiple factors, including the intrinsic deficiency of vitiligo melanocytes and exogenous stimuli [24]. The excessive accumulation of ROS disrupts the skin homeostasis, resulting in not only the increased sensitivity of melanocytes to external stimulus but also the inappropriate activation of the immune system that enables immune cells to attack and destroy melanocytes [25]. It has been demonstrated that oxidative stress disorder also plays a crucial role in the induction of chemokines, and cells capable of killing melanocytes, especially melanocyte-specific CD8+ T cells, are guided and migrate into the skin tissue in response to the chemotactic signals [10, 24]. As shown in Figure 1, the inducible chemokines-mediated interplay between oxidative stress and autoimmune response may be an important factor in the pathogenesis and progression of vitiligo.

### 3.1. CXCL16-CXCR6 Axis in Vitiligo

The chemokine ligand CXCL16 expressed on the cell surface can help cell-cell adhesion or function as a scavenger receptor, while the shedding of CXCL16, as a soluble molecule, is involved in chemotaxis of CXCR6-expressing cells [14, 26]. As gene analysis on cells isolated from lesional and nonlesional skin of vitiligo has identified CXCL16 and CXCR6 in the pathogenesis of the disease, the role of the CXCL16-CXCR6 axis in vitiligo is better appreciated in recent studies [27].

An early study revealed the constitutive expression of CXCL16 on epidermal keratinocytes in healthy skin, while cell activation or cellular stress such as UVB irradiation could promote the shedding of CXCL16, acting as a chemoattractant for the recruitment of T cells [26]. A more recent study noticed that the expression of CXCL16 in the serum of vitiligo patients was positively correlated with ROS level [10]. Based on this observation, researchers utilizing human keratinocytes found that ROS could induce the production of CXCL16 in vitro [28]. The ROS-induced CXCL16 expression was associated with the activation of untranslated protein response (UPR), partly through 2 UPR parallel signaling branches: the PRKR-like ER kinase (PERK)-eukaryotic translation initiation factor 2*α* pathway and the inositol-requiring enzyme-1–X-box-binding protein 1 (XBP1) pathway [10]. Under the promoted CXCL16 production, the cutaneous infiltration of CXCR6+ CD8+ T cells was increased in vitiligo perilesional skin, leading to melanocyte loss in vitiligo lesions. Blocking the CXCL16-CXCR6 axis significantly impaired the CD8+ T cell recruitment to the skin [10].

Oxidative stress can also promote CXCL16 production indirectly by inducing high-mobility group protein B1 (HMGB1), a damage-associated molecular pattern molecule, which further facilitates the migration of autoreactive T cells and the maturation of DCs [29]. The oxidative stress can also lead to the activation of nucleotide-binding oligomerization domain, leucine-rich repeat, and pyrin domain-containing protein 3 (NLRP3) inflammasome complex via transient receptor potential cation channel subfamily M member 2 [30, 31]. The dysregulation of NLRP3 has been found in patients with vitiligo, especially those with progressive disease. Of greater interest, researchers found that NLRP3 activation in stressed keratinocytes promoted CXCL16-CXCR6 chemotactic signaling through IL-1*β*/IL-1R signaling [32]. The involvement of NF-*κ*B and IRF1 pathways in the regulation of CXCL16 in keratinocytes under oxidative stress were also found [10, 32].

Recently, it has been reported that the virus infection, stimulated by Poly(I:C), can augment chemokine CXCL16 production. Mechanistically, the regulation of CXCL16 is mainly associated with activated intracellular virus sensor melanoma differentiation-associated 5 protein and mitochondrial antiviral signaling protein-mediated IRF3 and NF-*κ*B pathways [33].

Taken together, these studies reinforce the role of keratinocyte-derived CXCL16 in the activation of melanocyte-specific autoimmunity, especially in the context of oxidative stress, indicating that inhibition of this chemotactic axis may be a promising treatment option for vitiligo patients.

### 3.2. Interferon-*γ*- (IFN-*γ*-) CXCL9/CXCL10-CXCR3 Axis in Vitiligo

Studies focusing on the CXCL16-CXCR6 axis do not mean excluding the role of other chemokines in the pathogenesis of vitiligo. Although in the case of vitiligo, CD8+ T cells are critical for the melanocyte loss, more and more studies demonstrated the emerging role of imbalanced Th1 pattern in the development of vitiligo, accompanied by the altered production of type-1 cytokines IFN-*γ* [34, 35]. Genetic studies have identified susceptibility genes in vitiligo, among which the TSLP gene is closely associated with inflammatory cytokines. The deficiency of TSLP gene results in the dominance of the Th1 immune response and further activates the IFN-*γ*-CXCL10 axis [9]. Researchers using genome-wide profiling revealed the vitiligo blood transcriptomics and found involvement of STAT1/IRF9, which also reinforced the important role of the IFN-*γ*-chemokine axis [36]. Analysis of gene expression profiling revealed the elevations of IFN-*γ* and IFN-*γ*-induced genes in the serum and lesional skin from patients with vitiligo and mouse models of vitiligo, and researchers found that the IFN-*γ* levels were correlated with the progression and maintenance of vitiligo, indicating that IFN-*γ* could serve as a reliable risk factor in vitiligo patients [37–42]. In vitiligo mice, melanocyte-specific CD8+ T cells were a major source of IFN-*γ*, and circulating IFN-*γ*+ CD8+ cytotoxic T lymphocytes (CTLs) were also enriched in vitiligo patients [38, 39, 43]. A recent study found that IFN-*γ* derived from CD8+ CTLs could directly induce apoptosis in melanocytes, leading to the release of self-antigens [41]. Consistently, the elevated levels of IFN-*γ*-inducible chemokines CXCL9 and CXCL10 in the serum or skin samples from vitiligo patients were confirmed in further studies [44–46]. The noticeable upregulation of epidermal CXCL10 and CXCL9 in mice and human tissues was attributed to the stressed keratinocytes, as well as approximately 30% of T cells and Langerhans cells in the epidermis [47]. These two chemokines share a common cognate receptor named CXCR3. Compared with the patients with stable vitiligo, the patients with progressive disease had higher frequencies of both CXCR3+ CD8+ and CXCR3+ CD4+ T cells, suggesting a potential indicative role of CXCR3 in disease activity [34].

Mitra et al. have shown a positive correlation between the elevated levels of ROS and IFN-*γ* production [48]. In addition, Yang et al. revealed a similar positive correlation between H_2_O_2_ levels and the expression of CXCL10 [49]. As mentioned above, chemical exposures are known to be triggers of oxidative stress for vitiligo. Chemicals such as 4-tertiary butyl-phenol (4-TBP) can halt protein synthesis in the endoplasmic reticulum, which consequently elicit the UPR in melanocytes [50, 51]. In response to cellular stress or UPR activation, molecular chaperone heat shock protein 70 (HSP70) can be released by keratinocytes to initiate a subsequent autoimmune response. The interplay between inducible HSP70i and plasmacytoid dendritic cells (pDCs) promotes the production of type I interferon IFN-*α* [52]. Moreover, HSP70 can also potentiate the production of IFN-*γ* in stressed NK and the group 1 innate lymphoid cells in vitiligo. The upregulated levels of IFN-*γ*, in turn, promoted the expression of HSP70i, forming the HSP70i-CTL-IFN-*γ*-HSP70i positive feedback [24, 53]. Together with IFN-*γ*, IFN-*α* enhances the secretion of CXCL9 and CXCL10 from keratinocytes to amplify the infiltration of CXCR3-expressing immune cells in lesional skin [54, 55]. These studies have provided evidence for the crosstalk between oxidative stress and autoimmune response orchestrated by chemotactic signals interferon-CXCL9/10-CXCR3 axis.

The upregulation of CXCL9 was first detectable in the new lesion of vitiligo, followed by increased levels of CXCL10. While mice with severe diseases exhibited a lack of higher expression of CXCL9, suggesting that CXCL9 may act more as a primary signal for T cell recruitment in vitiligo [56]. Researchers utilizing a CXCL9-deficient mouse model of vitiligo observed a decreased number of premelanosome protein-specific (PMEL) CD8+ T cells in both the dermis and epidermis; however, the deficiency in CXCL9 did not reverse the disease or reduce depigmentation significantly in mice with established vitiligo, which further confirmed the role of CXCL9 as a “recruit” signal in positioning and recruiting T cells [56]. In contrary to CXCL9, CXCL10 has been demonstrated as tethering signals involved in localization and effector functions of T cells [35]. Additionally, there is evidence to show that exposure to CXCL10 can significantly induce human melanocyte apoptosis through the CXCR3B-mediated signal, leading to the release of self-antigens to initiate the adaptive immune responses [56, 57]. Blocking the CXCR3 expression inhibits CXCL10-induced apoptosis, and neither transferred CXCR3-deficient T cells nor CXCR3-depletion mice could induce pigmentation [56, 57].

Collectively, ROS accumulation following oxidative stress induces the production of chemokines, thus promoting the infiltration of T cells trafficking into vitiligo lesions, providing insight into the importance of the CXCL9/CXCL10-CXCR3 axis that orchestrates the migration of immune cells involved in the progression and maintenance of vitiligo, indicating potential targets for treating vitiligo.

### 3.3. CXCL8 In Vitiligo

Elevated CXCL8 serum levels and increased CXCL8 gene expression in vitiligo patients have been reported recently [58]. Likewise, another pilot study also documented the higher serum CXCL8 levels. Moreover, compared with stable patients, active vitiligo patients exhibited higher levels of CXCL8 in serum as well as in blister fluid of lesions [45]. Exposing melanocytes to chemical triggers, such as 4-TBP and monobenzone, oxidative stress can induce the production of IL-6 and CXCL8, and their expression is regulated partly by XBP1 during the activation of UPR [59]. A more recent study indicated that promoted release of HMGB1 could augment the secretion of CXCL8 via NF-*κ*B p65 and ERK pathways in response to oxidative stress [29].

Despite the detectable changes in CXCL8 levels in vitiligo, the well-known function of CXCL8 in mediating the migration of neutrophils rather than T cells is insufficient to elucidate the role of CXCL8 in the pathogenesis of vitiligo, because of the absence of neutrophils in vitiligo lesion skin [35]. Some studies reported that 8-hydroxydeoxyguanosine was increased in vitiligo under oxidative stress and inhibited the Rac-GTPases, as the neutrophil migration can also be mediated by Rac-GTPases, probably resulting in suppressing the migration of neutrophils in vitiligo even in the presence of elevated CXCL8 [29, 60, 61]. However, CXCL8 has also been proposed as a powerful chemokine to indirectly induce the apoptosis of melanocytes and keratinocytes through oxidative stress, and the apoptotic cells can release proinflammatory cytokines and chemokines, which may favor the cutaneous infiltration of immune cells in vitiligo [62, 63]. The functional role of CXCL8 and its corresponding receptors still warrants further studies.

### 3.4. Alternation on Other Chemotactic Molecules in Vitiligo

#### 3.4.1. CXCL12

The CXCL chemokine ligand, CXCL12, is a chemokine involved in many physiological and pathologic events, such as embryogenesis and tumorigenesis. The first described receptor for CXCL12 is CXCR4, which was first considered a homeostatic receptor. The expression of CXCR4 can also be modulated under inflammation conditions, serving as an important chemotactic signal for the recruitment and retention of leukocytes [64]. ACKR3 (CXCR7) is another high-affinity receptor for CXCL12; however, there is a lack of investigations into ACKR3 in vitiligo up to date [14].

Regarding the migration of immune cells toward the inflammatory sites, many studies highlight the importance of CXCL12/CXCR4 in autoimmune diseases, including psoriasis, rheumatoid arthritis, [64]. A recent study integrated genomics and proteomics to propose a series of potential drug targets and biomarkers, and CXCL12 was on the top of the listed secretary proteins in vitiligo [65]. The function of CXCL12 as an efficient predictor of vitiligo has also been confirmed in other studies. Compared with the healthy control, elevated CXCL12 was observed in the serum of vitiligo patients, with a higher secretion of CXCL12 in patients with progressive vitiligo. ROC curve pointed out a significant correlation between CXCL12 level and disease progression, especially in patients who appeared to be stable or even improved before recurrence [66]. Studies examined patient-derived melanocytes and skin samples, and found a significant increase in the expression of epidermal melanocyte-derived CXCL12, surrounded by CXCR4+ cells in early vitiligo [67]. It is noted that CXCR4 and CXCL12 have been recognized as critical mediators for mobilizing the IFN-producing cells and Langerhans cells in the skin [66, 68]. The in vivo animal studies further confirmed that CXCL12 enhanced the recruitment of APCs and T cells to the site's proximity to melanocytes, resulting in melanocyte destruction and pigmentation [67]. On the other hand, the prominent infiltration of CD11c+ CXCL12+ DCs was found in vitiligo-affected skin in the early stage of the disease. These DCs could promote the migration of CXCR4+ keratinocytes and the activation of epidermal T cells by assisting the acquisition of melanocyte antigens [69]. Although oxidative stress and hypoxia could enhance the CXCL12 production, when exposure to ER stress or upregulated ROS concentration, the expression of CXCL12 was not altered substantially, warranting more researches into the molecular mechanisms of its involvement in the onset and progression of vitiligo [64, 67].

#### 3.4.2. CCL22

Skin-homing of Tregs are supposed to exert their immunosuppressive function against autoreactive CD8+ T cells, and decreased frequency of Tregs was observed in vitiligo [70, 71]. Chemokine ligand-receptor pairs that guide the migration of Tregs include CCL1 and CCR8, CCL21 and CCR7, and CCL22 with its receptor CCR4, while studies on vitiligo skin samples only revealed a significant reduction in CCL22 expression [72, 73]. Although transcriptional analysis of vitiligo-associated gene profiles indicated a significant increase in CCL20 [74], in the mouse model of vitiligo, overexpression of CCL22 could promote the recruitment and regulatory function of Tregs, accompanied by a decreased abundance of melanocyte-specific effector T cells, and resulted in alleviating depigmentation and preventing vitiligo in mice. But a continued treatment was required for the long-term therapeutic effects [75]. A better understanding of Treg trafficking by chemoattractant molecules under oxidative stress may provide a potential therapeutic option for vitiligo treatment.

#### 3.4.3. CCL20

Increased levels of CCL20 were also reported in serum and vitiligous skin from patients with vitiligo, and CCL20 levels were significantly higher in patients with active vitiligo. Serum levels of CCL20 were positively correlated with VASI (Vitiligo Area Severity Index) and VETF (Vitiligo European Task Force), and decreased CCL20 expression was also found after the treatment [76], suggesting CCL20 as a reliable biomarker for the disease activity and an indicative marker for the therapeutic efficacy. Another chemokine ligand CCL19, which attracts CCR7+ cells, was involved in the development of vitiligo during the treatment of advanced melanoma by immune checkpoint inhibitors [77]. However, there is a paucity of studies investigating the functional roles of these chemoattractant molecules under excessive oxidative stress in the pathogenesis of vitiligo.

## 4. The Perspective of Targeted Therapies in Vitiligo

Current treatments for vitiligo include phototherapy, topical and systemic immunosuppressive agents, and surgical management, which require a long-term follow-up to assess therapeutic efficacy. Nevertheless, among these nontargeted treatment options, there is no such potent therapy that can induce complete repigmentation or durable therapeutic effects without recurrence, warranting more therapeutic approaches for the management of vitiligo [78].

### 4.1. Antioxidative Strategies in Vitiligo

Regarding the importance of the imbalance between excessive oxidative stress and depletion of endogenous antioxidants in the pathogenesis of vitiligo, it is increasingly evident that antioxidant supplementation alone or in combination with other conventional therapies may serve as a promising strategy for vitiligo patients [25].

Aside from the endogenous antioxidant system in the skin (e.g., superoxide dismutase (SOD), glutathione reductase, glutathione peroxidase, and enzymatic antioxidants), food intake is an important source of exogenous antioxidants, such as green tea, silymarin, and squalene. It is reasonable to assume that oral antioxidant supplements may serve as a convenient approach with a good safety profile [79]. Early in 2009, Elgoweini and El Din conducted a study to determine the additional benefit of oral vitamin E in combination with narrowband ultraviolet B phototherapy (NB-UVB) and found that 72.7% of patients in the combined therapy group achieved better repigmentation, while in patients who only received NB-UVB, it was 55.6% [80]. Likewise, the antioxidant supplement by phyllanthus emblica, khellin, Ginkgo biloba, polyunsaturated fatty acid, or carotenoids also increased the therapeutic efficacy of topic therapy or phototherapy for vitiligo patients [81–83]. For localized vitiligo, topical antioxidant agents can be another valuable choice. The active ingredients in topical antioxidant hydrogel including folic acid, sitosterol, and hyaluronic acid markedly improved the therapeutic effect of excimer light [84, 85]. However, due to a lack of larger scales or multicenter comparative trials, the exact efficacies of these antioxidant agents require further studies. Generally, antioxidant agents alone are unable to elicit significant repigmentation in most of the studies, but they are likely to provide additional benefits as adjunctive approaches to conventional treatments.

### 4.2. Targeting Chemokines and Corresponding Receptors in Vitiligo

Based on the results of the mentioned studies above, it is rational to hypothesize that targeting ROS-induced chemotactic molecules directly may yield more potent therapeutic effects without interrupting other effector factors. In one study, CXCL10-deficient mice exhibited lower levels of autoreactive T cells residing in the skin. Compared with CXCL9-/- mice, a higher ratio of CD69+ CD44lo effector memory T cells was observed in CXCL10-/- hosts. CD44 expression is important for the survival and activation of memory T cells; CXCL10 may also play a critical role in driving CD8+ memory T cells into the vitiligo epidermis [38, 56]. Consistent with these observations, CXCL10 neutralizing antibody alleviated depigmentation effectively and induced repigmentation after 4 weeks of treatment in mice with established vitiligo, whereas neutralization of CXCL9 showed no significant improvement [56]. On the other hand, inhibition of CXCR3 was reported to prevent the CXCL10-induced melanocyte apoptosis and T cell infiltration in the skin, and blocking their receptor CXCR3 by CXCR3 antibodies led to repigmentation in mice with established vitiligo [53, 57]. Furthermore, depleting antibody of CXCR3 was more effective than neutralizing antibody in reducing melanocyte-specific CD8+ T cells [57]. Currently, there are several anti-CXCL10 monoclonal antibodies and anti-CXCR3 molecule blockades that are being investigated in clinical trials for autoimmune diseases such as rheumatoid arthritis [37]; however, the therapeutic efficacies of these antagonists in vitiligo patients are currently unknown.

In addition, the neutralizing antibody of CXCL16 was also found to reduce CD8+ T cell migration. Compared with CXCR3 depletion, CXCR6-deficient CD8+ T cells exhibited more significant effects on T cell skin-homing capacity, but its combination with CXCR3 elimination or CXCL10 antibody did not reveal any addictive effects on T cell migration [10].

In addition to conventional treatment options, therapeutic skin trauma such as microneedling, punch grafting and CO_2_ fraction laser can cause extrinsic injury to induce wound healing process and result in repigmentation in vitiligo-affected skin. Despite the possible role of upregulated CXCL12 in vitiligo activity and progression, studies also indicate that CXCL12 may also participate in these therapeutic trauma-induced skin repigmentation [86]. The increased levels of CXCL12 were observed during the wound healing of excisional or burn injuries, especially in wound margins, and the activation of the CXCL12/CXCR4 axis lead to the accumulation of eosinophil and accelerate the neovascularization and epithelialization [87, 88]. A more recent study revealed that 5-fluorouracil (5-FU) significantly increased the CXCL12 level in the vitiligo skin, and blocking the CXCL12/CXCR4 pathway, in turn, inhibited melanocyte migration [89]. It is hypothesized that under proper injury, the CXCL12-enriched microenvironment may favor melanocyte recruitment by trafficking corresponding melanocyte precursors into the perilesional and lesional vitiligo areas to induce therapeutic effects.

Notably, the IFN-*γ*-JAK-STAT signaling pathway is critical for CXCL10 production. The activation of STAT1 leads to the transcription of IFN-*γ*-inducible genes including CXCL10. Therefore, inhibiting the JAK-STAT pathway to interfere with CXCL10 signaling may be a promising approach for vitiligo. To date, the FDA-approved indications for JAK inhibitors (JAKi) include rheumatoid arthritis, myelofibrosis, and polycythemia vera, and there are no approved JAKi in vitiligo [90]. Considering efficacy in these autoimmune or autoinflammatory conditions, recent case reports and case studies reveal the therapeutic effects of JAKi in patients with vitiligo. A case report described a rapid regression of vitiligo that occurred in a patient treated with a potent JAK1/2 inhibitor ruxolitinib, and the reduced CXCL10 levels were observed after the treatment [91]. Another JAK1/3 inhibitor, tofacitinib citrate, induced a rapid and nearly complete repigmentation in a female patient with widespread and progressive vitiligo [92]. However, a relapse of depigmentation in the previously repigmented skin was also reported after stopping the JAKi treatment [91]. Subsequently, researchers further examined immune cells in a mouse model treated with JAKi and found that JAKi successfully induced significant repigmentation and reduced melanocyte-specific T cells in the epidermis. On the other hand, in the reversal model, autoreactive resident memory T cells were not markedly affected by JAKi treatment and could recruit autoimmune T cells into the skin again, which may partly explain the recurrent depigmentation in the case report [93]. Currently, there are three completed clinical trials (NCT03762551, NCT04057573, and NCT04052425) and five ongoing clinical studies of JAKi in vitiligo (NCT03185312, NCT04896385, NCT03099304, NCT04927975, and NCT04246372). A recent article reviewed 10 clinical studies in vitiligo on the use of JAKi, including six multicenter studies [94]. In studies examining the percentage decrease in body surface area, 50% of patients experienced positive therapeutic effects in facial repigmentation or body repigmentation, especially in sun-exposed areas. Vitiligo Area Scoring Index (VASI) was mainly considered in another three studies (one observational and two experimental), and these studies found that 70% of patients treated with ruxolitinib and 68% of patients treated with tofacitinib showed different degrees of improvement in VASI score [94]. The therapeutic effect of a topical formulation of JAK inhibitor has been examined in a small cohort of patients with vitiligo. Significant improvement in facial vitiligo with manageable adverse effects was observed in one open-label trial for the use of topical ruxolitinib [92]. As blocking autoimmune response combined with melanocyte stimulation may be more effective, this study was subsequently extended to investigate the synergism of concomitant administration of JAK inhibitor and NB-UVB treatment, and showed a statistically significant improvement in VASI [95]. Better repigmentation rates by tofacitinib plus concomitant NB-UVB were also obtained in several studies [96–98], one of which suggested that the vitiligo treatment by JAK inhibitor may require low-dose light exposure including NB-UVB [97]. Instead of the standard treatment regimen, the suppression of pathologic immune responses allows lower levels of UV irradiation that may be sufficient for vitiligo treatment with decreased risk of skin cancers. Despite the limited numbers of case reports and a lack of larger cohort studies, they still provide encouraging preliminary evidence for the monotherapy and combination therapy of JAK inhibitors, and further studies are still needed.

## 5. Conclusion

In response to oxidative stress and other stimulatory factors, chemokines are not only involved in the migration of innate immune cells but also determinate in adaptive immune cell trafficking. More and more studies have expanded the knowledge that these chemoattractant molecules are responsible for the initiation and progression of vitiligo. In this review, we provide evidence to indicate the aberrant production of chemokines through oxidative stress and activated autoimmunity. Among these chemokines, some are proven as reliable biomarkers for disease severity and prognosis after the treatment of vitiligo, and preclinical studies also provide insight into the potential therapeutic intervention by targeting the chemoattractant signals. A better understanding of the role of chemokines in vitiligo is needed for the development of promising therapies.
